# Evaluation of High Resolution Melting for *MTHFR* C677T Genotyping in Congenital Heart Disease

**DOI:** 10.1371/journal.pone.0151140

**Published:** 2016-03-18

**Authors:** Ying Wang, Haiyan Zhang, Shuying Yue, Kun Zhang, Hui Wang, Rui Dong, Xiaomeng Yang, Yi Liu, Yanhui Ma

**Affiliations:** 1 Research Institute of Pediatrics, Qilu Children’s Hospital of Shandong University, Ji’nan, 250022, China; 2 Department of Medicine, Shandong Medical College, Ji’nan, 250002, China; 3 Cardiovascular Department, Qilu Children’s Hospital of Shandong University, Ji’nan, 250022, China; University of Innsbruck, AUSTRIA

## Abstract

**Background:**

High resolution melting (HRM) is a simple, flexible and low-cost mutation screening technique. The methylenetetrahydrofolate reductase (MTHFR) gene encoding a critical enzyme, potentially affects susceptibility to some congenital defects like congenital heart disease (CHD). We evaluate the performance of HRM for genotyping of the *MTHFR* gene C677T locus in CHD cases and healthy controls of Chinese Han population.

**Methods:**

A total of 315 blood samples from 147 CHD patients (male72, female 75) and 168 healthy controls (male 92, female 76) were enrolled in the study. HRM was utilized to genotype *MTHFR* C677T locus of all the samples. The results were compared to that of PCR-RFLP and Sanger sequencing. The association of the *MTHFR* C677T genotypes and the risk of CHD was analyzed using odds ratio with their 95% confidence interval (CIs) from unconditional logistic regression.

**Results:**

All the samples were successfully genotyped by HRM within 1 hour and 30 minutes while at least 6 hours were needed for PCR-RFLP and sequencing. The genotypes of *MTHFR* C677T CC, CT, and TT were 9.52%, 49.66%, and 40.82% in CHD group but 29.17%, 50% and 20.83% in control group, which were identical using both methods of HRM and PCR-RFLP, demonstrating the sensitivity and specificity of HRM were all 100%.

**Conclusion:**

*MTHFR* C677T is a potential risk factor for CHD in our local residents of Shandong province in China. HRM is a fast, sensitive, specific and reliable method for clinical application of genotyping.

## Introduction

Congenital heart disease (CHD) is the most common defects with 1% prevalence worldwide and leading non-infectious cause of morbidity and mortality in infant [1]. It is generally agreed that both genetic and environmental factors are involved in the aetiology of CHD though the causes have not been fully understood [2,3]. Some researches reported that the methylenetetrahydrofolate reductase (MTHFR) gene is one of the most important susceptibility genes for CHD in different populations [4–10]. The enzyme MTHFR catalyzes the conversion of 5,10-methylenetetrahydrofolate into 5-methyltetrahydrofolate which is an critical precursor in methylation reactions and the changes of MTHFR activity thus influence both DNA methylation and synthesis. Recently, the *MTHFR* gene rs1801131 (C677T) has been identified as a risk factor for CHD in Asian, especially in Chinese Han population by using PCR-RFLP or PCR-sequencing [11–16]. High resolution melting (HRM) is a simple, rapid, flexible, sensitive, specific and low-cost mutation screening technique without post-PCR processing, has been strongly suggested as a standard approach for mutation scanning in clinical diagnosis [17,18].

In this study, we utilized HRM to genotype *MTHFR* gene C677T (rs1801133) in CHD patients and controls in Chinese Han population. The performance of this technique for the genotyping was evaluated in comparison with the method of PCR-RFLP and confirmed by PCR-sequencing.

## Materials and Methods

### Ethics statement

The work was approved by Ethics Committee of Qilu Children’s Hospital of Shandong University. Informed written consent was obtained from the guardians of patients. The patients’ information was anonymized prior to submission. All the procedures performed in the study were in accordance with the Declaration of Helsinki.

### DNA samples

A total of 147 unrelated children with congenital heart disease (CHD) (n = 147, Male:72, Female:75, average age: 1.46±1.91 years) and 168 healthy children (n = 168, Male:92, Female:76, average age: 3.08±0.86 years) as normal control were collected from June in 2013 to May in 2014. All participants were from Han Chinese population of Shandong Province and recruited from Qilu Children’s Hospital of Shandong University. The echocardiogram or cardiac catheterization was performed to estimate their cardiac status in CHD group. Only the patients with non-syndromic heart defects were included. Congenital heart defects in the cohort included atrial septal defect (ASD), ventricular septal defect (VSD), patent ductus arteriosus (PDA), patent foramen ovale (PFO), pulmonary arterial hypertension (PAH), aortic valve stenosis, coarctation of the aorta, pulmonary stenosis, transposition of the great arteries, tetralogy of Fallot, truncus arteriosus, tricuspid regurgitation, and ebstein anomaly. The normal control group was comprised of healthy children without past or present history of congenital defects and psychiatric conditions. Peripheral blood samples were obtained from both groups. Genomic DNA was extracted using TIANamp Blood DNA kit (TIANGEN, Beijing, China) following the manufacturer’s instructions.

### *MTHFR* C677T genotyping by high-resolution melting analysis

High-resolution melting of *MTHFR* C677T genotyping was performed with LightMix^®^ assay kit in a LightCycler 480 IImachine (Roche Diagnostics, Mannheim, Germany). The sequences of primer set were 5’-CTTTGAGGCTGACCTGAAGC-3’ (forward) and 5’-AGGACGGTGCGGTGAGAGTG-3’(reverse) which were reported before [19]. The optional parameters of 20μl real-time PCR reaction system included: 30ng of genomic DNA, 0.5μl of 1μM of each primer, 10μl 2 × LightCycler 480 High Resolution Melting Master Mix (containing FastStart Taq DNA Polymerase, reaction buffer, deoxynucleoside triphosphate [dNTP] mix, and High Resolution Melting Dye), 2μl of 2.5 mM MgCl_2_. Real-time PCR was run by an initial denaturation at 95°C for 10 min, and followed by 45 cycles of 95°C for 10 s, 64°C for 15 s, 72°C for 8 s. To determine the melting points, the melting curve analysis was performed at 95°C for 1 min and cooled to 40°C, followed by a slow heating from 65°C to 95°C at the rate of 1°C/s. Melting curve was analyzed with the Light-Cycler^®^ 480 Gene Scanning software version 1.2 (Roche Diagnostics, Mannheim, Germany). The normalization settings and reference genotype were exactly the same for each experiment. Each sample was performed three times in this study.

### *MTHFR* C677T genotyping by PCR-RFLP

Polymerase chain reaction—restriction fragment length polymorphism (PCR-RFLP) was utilized to genotype *MTHFR* C677T described by Dong et al [20]. The PCR primers were designed using an online primer designing tool—Primer 3 (http://primer3.ut.ee/) and sequences were: 5’-GCCTCTCCTGACTGTCATCC-3’ (forward) and 5’-AGGACGGTG CGGTGAGAGTG-3’ (reverse). According to *MTHFR* C677T polymorphism locus, digestion products can be divided into three genotypes under gel imaging system: wild type CC (282 bp), heterozygous mutation type CT (282 bp, 176 bp and 106 bp) and homozygous mutant TT (176 bp, 106 bp).

PCR amplification was carried out in a 25ul reaction volume, containing 30ng of genomic DNA, 0.4μM of each primer, 0.2mM dNTP, 2.5μl 10 x buffer, and 0.5U Taq DNA Polymerase in a DNA Engine Tetrad Thermal Cycler (MJ Research, San Francisco, CA, USA) and the cycling conditions included an initial denaturation at 95°C for 5min, followed by 35 cycles of 30 sec at 95°C, 30 sec at 65°C, and 30 sec at 72°C, a final extension step at 72°C for 5min. After amplification, 8 μl of the PCR products were mixed and digested with 1μl (10000U/ml) Hinf I (New England BioLabs, Ipswich, USA) and 1μl 10 x buffer and then incubated for 3 hours at 37°C. Digestion products were separated by 2.5% agarose gel electrophoresis and observed by gel imaging system (Tanon 2500, Shanghai, China).

Twenty PCR products from both groups of samples were randomly selected to perform Sanger sequencing in a 3730 DNA Analyzer (Life Technologies, Grand Island, NY, USA) and verify the results of PCR-RFLP.

### Statistical Analysis

The performance of HRM for the genotyping of *MTHFR* G677T was evaluated by comparing with PCR-RFLP/PCR-sequencing. The chi-square (χ^2^) test was used for deviation from Hardy–Weinberg equilibrium, and comparison of the allelic and genotype frequencies between CHD groups and controls. The associations between the *MTHFR* genetic polymorphisms and the risk of CHD were analyzed using odds ratio with their 95% confidence interval (CIs) from unconditional logistic regression analyses. Statistical analysis was performed with SPSS 16.0 software. It was considered statistically significant when *P* value was less than 0.05 and the confidence interval was 95%.

## Results

### General distributions of the population

The *MTHFR* C677T genotype data were obtained from 147 CHD cases and 168 control samples. The genotype distributions were all within Hardy–Weinberg equilibrium.

### Sensitivity and specificity of HRM genotyping

**Fig 1:**
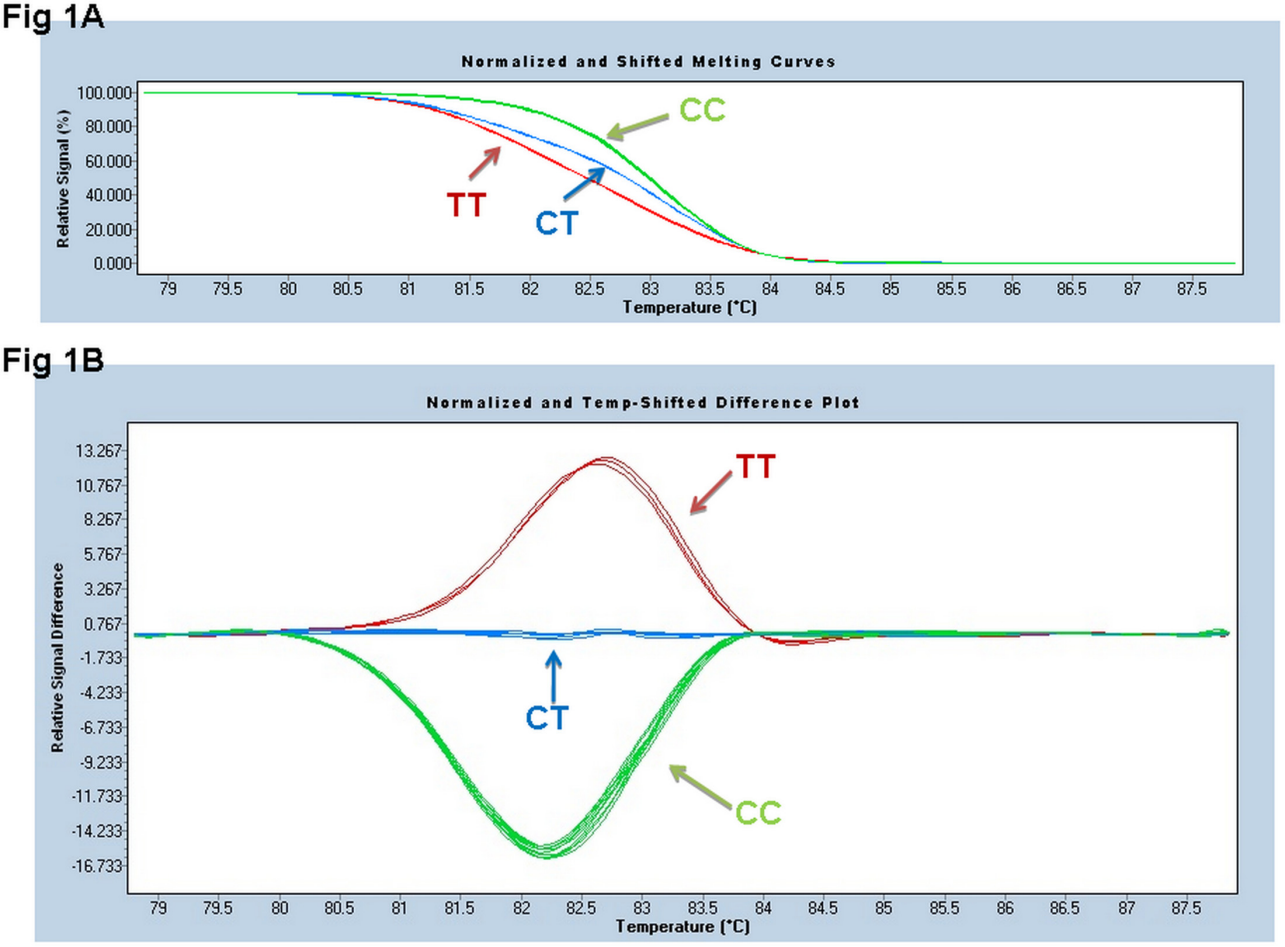
High-resolution melting analysis of a *MTHFR* gene fragment containing C677T polymorphism. The wild type CC was used as a base line in the Normalized and shifted melting curves (Fig 1A) as well as the Temp-Shifted Difference Plot (Fig 1B). Wild-type (CC) is presented in green, heterozygous mutant in blue (CT) and homozygous mutant is in red (TT).

**Fig 2:**
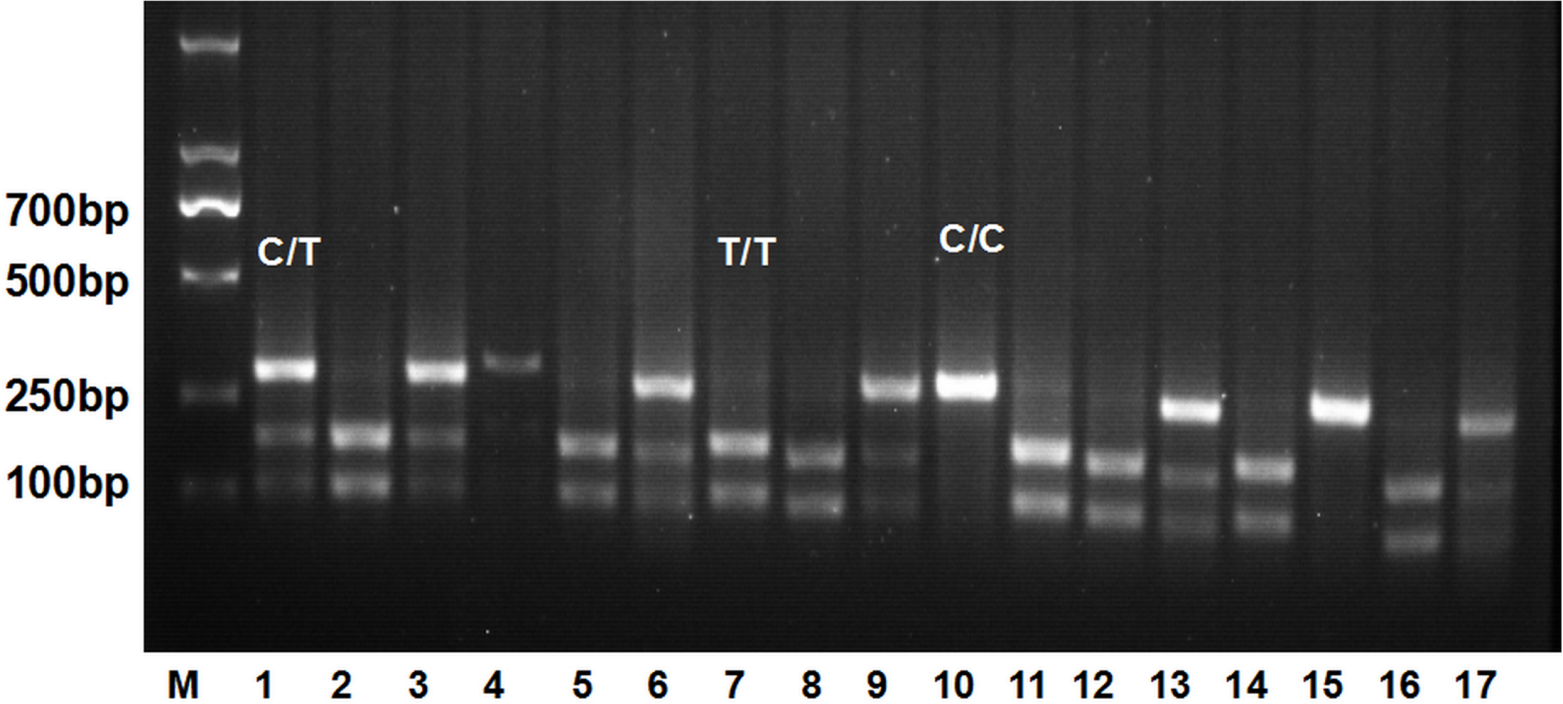
PCR-RFLP results of MTHFR C677T polymorphisms. Lane 1 is DNA marker. Lane 4, 10 and 15 are wild type genotype (CC). Lane 1, 3, 6, 9, 13, and 17 are heterozygous mutant genotype (CT). Lane 2, 5, 7, 8, 11, 12, 14 and 16 are homozygous mutant genotype (TT).

All the samples were successfully genotyped with HRM technique within only 1 hour and 20 minutes while 6 hours had to be taken for PCR-RFLP. The normalized melting curves of 2 kinds of *MTHFR* mutations (CT, TT) were readily distinguished from the wild-type (CC) (Fig 1). The genotypes of *MTHFR* C677T (CC, CT and TT) were 20%, 49.84% and 30.16%, which were identical as that of PCR-RFLP/PCR-sequencing and no false and errors were observed. The sensitivity and specificity reached 100%. The representative results of PCR-RFLP from 17 samples were shown in Fig 2.

### Genotyping and frequencies of *MTHFR* genetic polymorphisms

The genotypes of *MTHFR* C677T CC, CT, and TT were 9.52%, 49.66%, and 40.82% in CHD group but 29.17%, 50% and 20.83% in control group, respectively. The genotype distributions and allelic frequencies for *MTHFR* C667T polymorphisms among all CHD cases and control samples were displayed in Table 1.

**Table 1:** *MTHFR* C677T distribution in CHD and control groups.

Groups	Genotype frequencies(%)			Allele frequencies(%)	
	CC	CT	TT	C	T
**CHD patients (n = 147)**	14 (9.52)	73 (49.66)	60 (40.82)	101 (34.35)	193 (65.65)
**Non-CHD controls (n = 168)**	49 (29.17)	84 (50)	35 (20.83)	182 (54.17)	154 (45.83)
**Total (n = 315)**	63 (20)	157 (49.84)	95 (30.16)	283 (44.92)	347 (55.08)
	χ^2^ = 25.507,*P*< 0.01			χ^2^ = 24.878,*P<* 0.01	

The distribution of *MTHFR* genotypes in different types of CHD was analyzed separately as presented in Table 2. The significant differences were observed in overall CHD and type-dependent genotype distribution as well as allele frequencies between patients and control (Tables 1 and 2). Owing to small numbers of samples, aortic valve stenosis, coarctation of the aorta, pulmonary stenosis, transposition of the great arteries, tetralogy of Fallot, truncus arteriosus, tricuspid regurgitation, and ebstein anomaly, were not analyzed and counted, individually.

**Table 2:** *MTHFR* C677T genotype frequencies in the CHD group at the type level.

Type of CHD	Genotype frequencies (%)				Allele frequencies (%)			OR (95% CI)				
	CC	CT	TT	χ2 value (*P*)	C	T	χ2 value (*P*)	TT vs CC	CT vs CC	TT/CT vs CC	TT vs CT/CC	T vs C
**All CHD (n = 147)**	14 (9.52)	73 (49.66)	60 (40.82)	25.51**	101 (34.35)	193 (65.65)	25.51**	6 (2.9–12.4)	3.04 (1.55–5.95)	3.91(2.06–7.44)	2.62(1.60–4.31)	2.26(1.64–3.12)
**VSD (n = 79)**	11 (13.92)	34(43.04)	34 (43.04)	15.17**	56 (35.44)	102(64.56)	15.17**	4.33 (1.9–9.7)	1.8 (0.84–3.88)	2.55 (1.241–5.22)	2.87 (1.61–2.131)	2.15 (1.46–3.18)
**ASD (n = 37)**	4 (10.8)	19 (54.4)	14 (37.8)	7.63*	182 (54.17)	154 (45.83)	7.63**	4.9 (1.49–16.15)	2.77 (0.89–8.62)	3.4 (1.14–10.1)	2.31 (1.08–4.95)	2.06 (1.22–3.46)
**PDA (n = 37)**	1 (2.7)	22 (59.5)	14 (37.8)	12.89*	24 (32.4)	50 (67.6)	11.46**	19.6 (2.46–156.04)	12.83 (1.68–98.18)	14.82 (1.98–111.15)	2.31 (1.08–4.95)	2.46 (1.45–4.19)
**PFO (n = 33)**	1 (3.0)	19(57.6)	13(39.4)	11.86**	21 (31.8)	45 (68.2)	11.02**	18.2 (2.27–145.64)	11.08 (1.44–85.37)	13.18 (1.75–99.13)	2.47 (1.12–5.45)	2.53 (1.45–4.44)
**PAH (n = 57)**	5 (8.8)	31 (54.4)	21 (36.8)	11.92**	41(36)	73 (64)	11.02**	5.89 (2.02–17.1)	3.62 (1.32–9.91)	4.28 (1.61–11.37)	2.22 (1.15–4.27)	2.1 (1.36–3.26)
**Others (n = 48)**	4 (8.3)	26(54.2)	18(37.5)	10.96**	34(35.4)	62 (64.6)	24.88**	6.3 (1.96–20.24)	3.79 (1.25–11.51)	4.53 (1.54–13.29)	2.28 (1.14–4.56)	2.16 (1.35–3.45)

*: 0.01<*P*<0.05;

**: *P*<0.01. CHD: congenital heart defects, VSD: ventricular septal defect, ASD: atrial septal defect, PDA: patent ductus arteriosus, PFO: patent foramen ovale, PAH: pulmonary arterial hypertension, CI: confidence interval, OR: odds ratio, *P*: calculated p-value by χ2 test.

We estimated the potential associations between the *MTHFR* C667T genetic polymorphisms and the risk of CHD by adjusted odds ratio and their 95% CIs from logistic regression analyses, with adjustment for age, although the small number of patients in the study. The data showed that the *MTHFR* C667T genetic polymorphisms was significantly associated with the increased risk for all CHD cases in the homozygote comparison (TT versus CC: OR = 6, 95% CI = 2.9–12.4), heterozygote comparison (CT versus CC: OR = 3.04, 95% CI = 1.55–5.95), dominant model (TT/CT versus CC: OR = 3.91, 95% CI = 2.06–7.44), recessive model (TT versus CT/CC: OR = 2.62, 95% CI = 1.60–7.44), and allele comparison (T versus C: OR = 2.26, 95% CI = 1.64–3.12) as presented in Table 2.

## Discussion

In this study, we used HRM to analyze *MTHFR* C677T genotypes in 315 samples and validate the result via PCR-RFLP/PCR-sequencing. The *MTHFR* C677T genotyping of all the samples was performed successfully by two methods and showed completely consistent result with 100% sensitivity and specificity. The significant differences were observed in the distribution of C677T genotypes in both groups (*P*<0.01). The frequency of T allele (193) in the cases was significantly higher than that (154) in controls (*P*<0.01).

It is known that MTHFR, encoded by *MTHFR* gene, is a critical enzyme involved in the folate/homocysteine metabolic pathway. *MTHFR* gene is located at chromosome 1p36.22, spanning 20.37 Kb of the genomic DNA and including 12 exons. Genetic variation in this gene affects susceptibility to neural tube defects, colon cancer and neurodevelopmental disorders. *MTHFR* gene polymorphism has also been reported to be associated with CHD in different populations, such as American, Caucasian, Egyptian, Portuguese, Puerto Rican, Mexican, Turkish and so on[4–10]. Recently, the *MTHFR* gene rs1801131 (C677T) has been identified as a risk factor for CHD in Chinese Han population with PCR-RFLP technique [11–16]. The substitution of C to T at position 677 (C677T) is the most common variant of the gene and has been proved to reduce MTHFR enzyme activity due to amino acid changing from alanine to valine [21]. In the study, the potential association between the *MTHFR* C677T and the risk of CHD was noticed by adjusting odds ratio and the 95% CIs, demonstrating the significantly association with the increased risk for our CHD cases.

The genotype of *MTHFR* C677T used to be analyzed by polymerase chain reaction—sequencing or restriction fragment length polymorphism (PCR-RFLP) techniques, which have been currently the most common methods for genotyping with requirement of post-PCR processing. PCR-sequencing known as “gold standard” for genotyping and mutation detection, was established by Sanger in 1977 [22] and is a preferred method due to its high reliability and robustness, but it takes longer time after regular PCR. PCR-RFLP based on the creation or deletion of recognition site of a restriction endonuclease by nucleotide variations in the polymorphic site, is a simple, reliable, relative fast and inexpensive method in comparison with PCR-sequencing. It was first reported in 1989 after the emergence of PCR [23] with obvious shortcoming such as long digestion time and availability of proper restriction enzyme(s). PCR product containing *MTHFR* C677T locus could be directly sequenced in 24 to 48 hours or digested into three genotypes of wild type CC, heterozygous mutant CT and homozygous mutant TT by endonuclease Hinf I, which usually takes over 6 hours. High resolution melting (HRM) technique recently developed as a closed-tube method with new ‘saturation’ dyes such as LCGreen, is becoming a powerful technique and has been well documented on many platforms and considered an ideal method for detecting DNA variants, without sample processing or separations after PCR. Theoretically, HRM can be used to detect any novel genotypes with the capability of differentiating the double-stranded DNA from the single-stranded DNA by imperceptible changes in fluorescence intensity. The novel genotypes were inferred from changes in the melting temperature and plot shapes of the PCR amplicons. Norambuena et al. [19] designed a small amplicon using common *MTHFR* variants as a model to validate the technique of HRM for screening DNA mutations. It was shown that this assay was very fast with only 1.5 hours and cost effective, which are particularly important for a routine diagnostic setting [17–19]. We compared the three common genotyping methods of HRM, PCR-RFLP and sequencing at various aspects, such as processing time, hands-on time, operational ease, processing steps, reproducibility, and summarized in Table 3.

**Table 3:** Comparison of genotyping methods among HRM, PCR-RFLP and sequencing.

Methods	HRM	PCR-RFLP	PCR-sequencing
Processing time	1 .5 h	> 6 h	> 24 h
Hans-on-time	0.5 h	2 h	2 h
Simplicity	Simple	complicated	Tedious
Processing steps	real-time PCR	regular PCR, digestion, electrophoresis	regular PCR and sequencing
reproducibility	good	good	Good
Accuracy	accurate	accurate	Gold standard
Cost and suitability	low, suitable for large samples	lower than HRM, adapted to small samples	high, adapted to small samples

In this study, we’ve performed the diagnostic validation of HRM for genotyping of *MTHFR* C677T in Chinese Han population. *MTHFR* C677T alone is a potential risk factor for CHD in our local residents of Shandong province in China. HRM is not only simple, time-saving, but highly sensitive, specific and reproducible for clinical diagnosis of genotyping.
